# Validating earliest rice farming in the Indonesian Archipelago

**DOI:** 10.1038/s41598-020-67747-3

**Published:** 2020-07-03

**Authors:** Zhenhua Deng, Hsiao-chun Hung, Mike T. Carson, Adhi Agus Oktaviana, Budianto Hakim, Truman Simanjuntak

**Affiliations:** 1 Center for the Study of Chinese Archaeology, Peking University, Beijing, 100871 China; 2 School of Archaeology and Museology, Peking University, Beijing, 100871 China; 3 Department of Archaeology and Natural History, Australian National University, Canberra, ACT 2061 Australia; 4 Micronesian Area Research Center, University of Guam, Mangilao, Guam 96913 USA; 5 Center for Prehistoric and Austronesian Studies, and National Center for Archaeology, Jalan Raya Condet Pejaten 4, Jakarta, 12510 Indonesia; 6 Balai Arkeologi Makassar, Jl. Pajjaiang No.13, Sudiang Raya, Kota Makassar, Sulawesi Selatan 90242 Indonesia

**Keywords:** Archaeology, Plant domestication

## Abstract

Preserved ancient botanical evidence in the form of rice phytoliths has confirmed that people farmed domesticated rice (*Oryza sativa*) in the interior of Sulawesi Island, Indonesia, by at least 3,500 years ago. This discovery helps to resolve a mystery about one of the region’s major events in natural and cultural history, by documenting when rice farming spread into Indonesia, ultimately from a source in mainland China. At the Minanga Sipakko site in Sulawesi, preserved leaf and husk phytoliths of rice show the diagnostic morphology of domesticated varieties, and the discarded husks indicate on-site processing of the crops. The phytoliths were contained within an undisturbed, subsurface archaeological layer of red-slipped pottery, a marker for an evidently sudden cultural change in the region that multiple radiocarbon results extend back to 3,500 years ago. The results from Minanga Sipakko allow factual evaluation of previously untested hypotheses about the timing, geographic pattern, and cultural context of the spread of rice farming into Indonesia, as well as the contribution of external immigrants in this process.

## Introduction

Rice is one of the most important staple crops in the world today, feeding more than one third of the human population^1^. The Indonesian Archipelago, and more broadly Island Southeast Asia (ISEA), is one of the main regions of rice cultivation and consumption, with a population of more than 267 million people. With roots around 9,000 years before present (ybp) in the middle and lower regions of the Yangtze Basin of China^2–4^, rice-farming traditions expanded during the next several millennia into many areas of East and Southeast Asia, through variable pathways and contexts^5–7^. However, debates about the origins, timing, and contexts of ancient rice farming in ISEA often have occurred without the benefit of archaeo-botanical analysis^8–12^. These questions have been especially persistent in Indonesia, but now we can report for the first time a long record of rice farming in Sulawesi.

Because of poor organic preservation in the humid tropics of ISEA, until now only very few occurrences of ancient rice remains have been found, for example at two sites with directly dated rice husks inside ancient pottery fabrics. These cases alone do not constitute sufficient evidence to differentiate domesticated versus wild rice. One of these discoveries was of a complete rice grain in a potsherd directly dated by C14 to 4,807–3,899 ybp (CAMS 725)^13^, from Gua Sireh in Sarawak, Malaysian Borneo, but this instance has been re-assessed as possibly a grain of wild rice that was incorporated by natural processes into the clay used for making the pottery^14^. Another direct C14 date of 3,975–3,380 (3,400 ± 125) ybp came from a single rice husk found inside pottery at Andarayan, northern Luzon, Philippines^15^. Other more recent studies have reported charred rice grains in archaeological deposits close to 3,000 ybp in northern Luzon^16^, and additional rice phytolith evidence has been reported previously from Minanga Sipakko and the nearby site of Kamassi in Sulawesi^17^.

**Figure 1:**
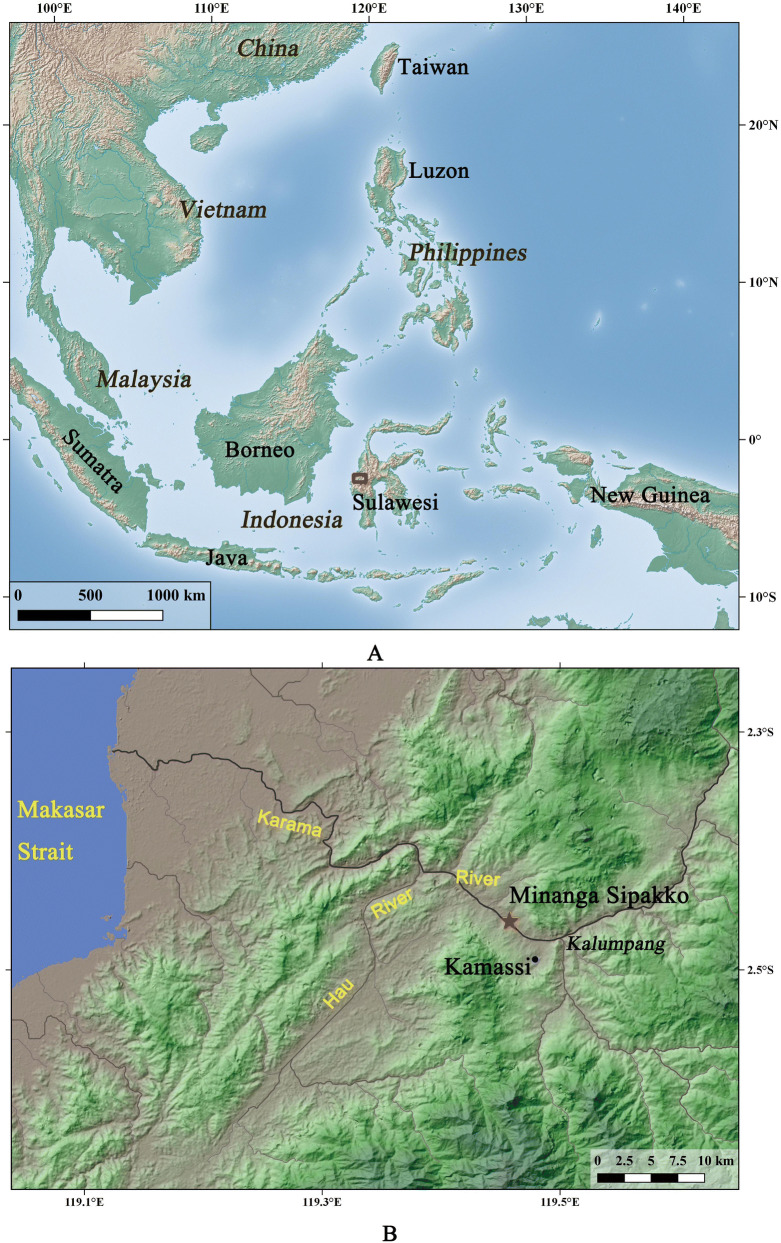
(**A**) Location of the research area (red rectangle) in Sulawesi and the broader region; (**B**) Setting of the Minanga Sipakko site, near Kalumpang township in the Karama Valley, West Sulawesi Province, Indonesia (Maps generated by Z. Deng, using Qgis version 3.4.14).

In order to answer the key question of when rice farming first appeared in Indonesia, the present study examined the early pottery-bearing layers of the Minanga Sipakko site (S2°26′24″, E119°26′31″), situated on an alluvial terrace directly adjacent to the Karama River in West Sulawesi Province (Fig. 1). Discovered in 1949^18^, this site was excavated several times from 1994 to 2007^19–22^, especially in 2004–2005 when seven 1.5 by 1.5 m squares (M1–M5 and M8–M9) were excavated. The primary archaeological deposit has been described as a single horizontal unit within the alluvial terrace, situated between older and younger layers of the natural alluvium of the Karama River.

In June 2017, the non-backfilled excavation trench profiles from 2004 to 2005 were cleaned by our team, allowing the collection of 22 point-specific phytolith samples from securely defined positions within the stratigraphic layers. From those samples, microscopic examination enabled the identification of preserved phytoliths of rice and other botanical taxa, as well as charcoal particles that could be submitted for direct radiocarbon dating.

## Results

### Refining the site chronology and cultural sequence

Previous excavations dated Minanga Sipakko rather broadly, possibly as old as 3,800 ybp or as late as 2,500 ybp^22^. In the lowest and oldest portion of the archaeological layer, the pottery fragments came mostly from red-slipped thin-walled earthenware vessels, resembling the oldest pottery horizon seen in the Philippines and connected with origins in Taiwan^17,23–25^. These findings suggested that Minanga Sipakko contained one of the earliest pottery assemblages in the Indonesian Archipelago, and therefore the site was considered ideal for this current research.

**Figure 2:**
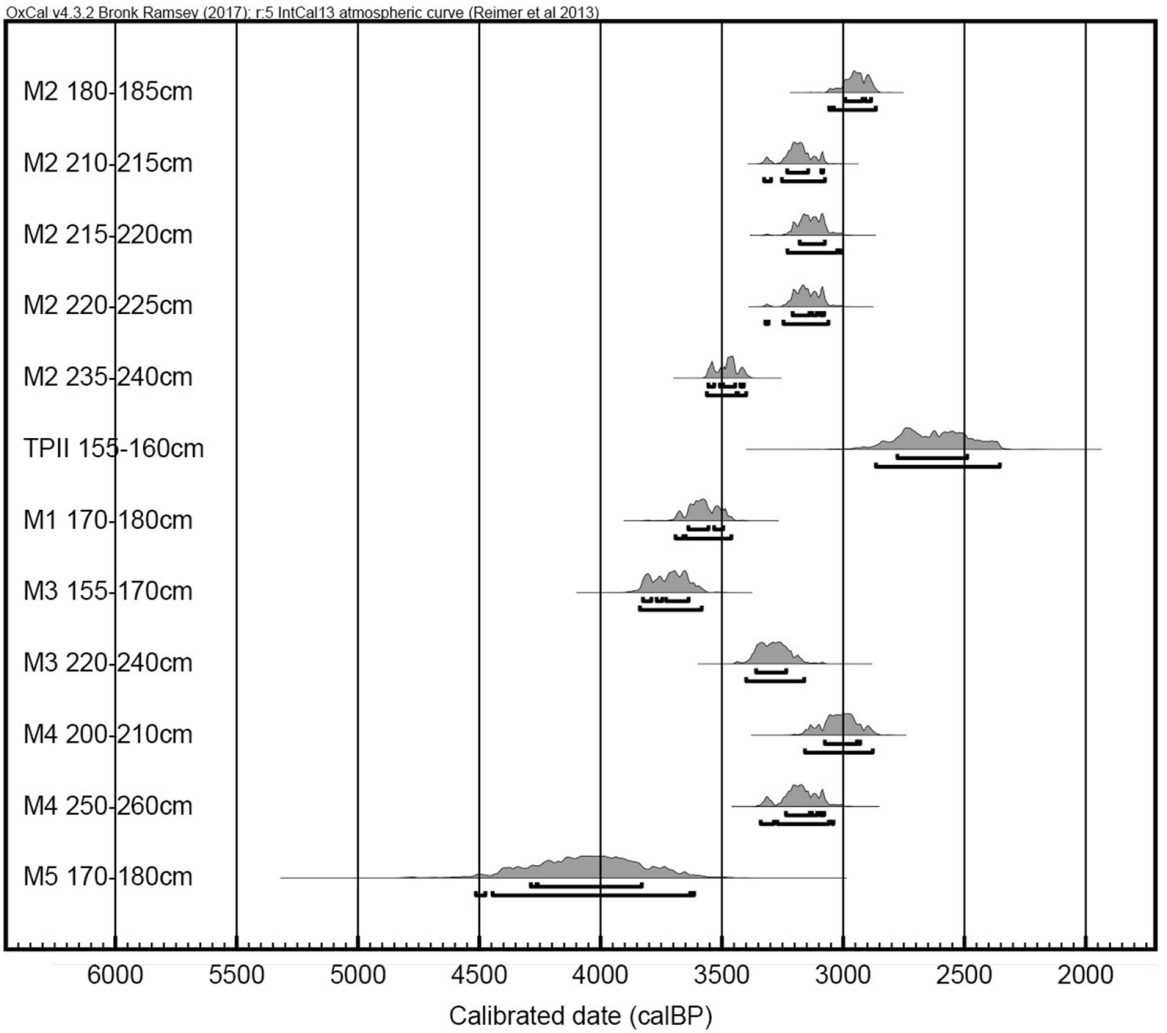
Calibrated radiocarbon dates from Minanga Sipakko. All dates for square M2 come from this study, and the others are from previously published reports^22^ (All dates calibrated by Z. Deng with OxCal v4.2.4^51^, using the IntCal13 atmospheric curve^52^ and presented with 2σ probability).

**Figure 3:**
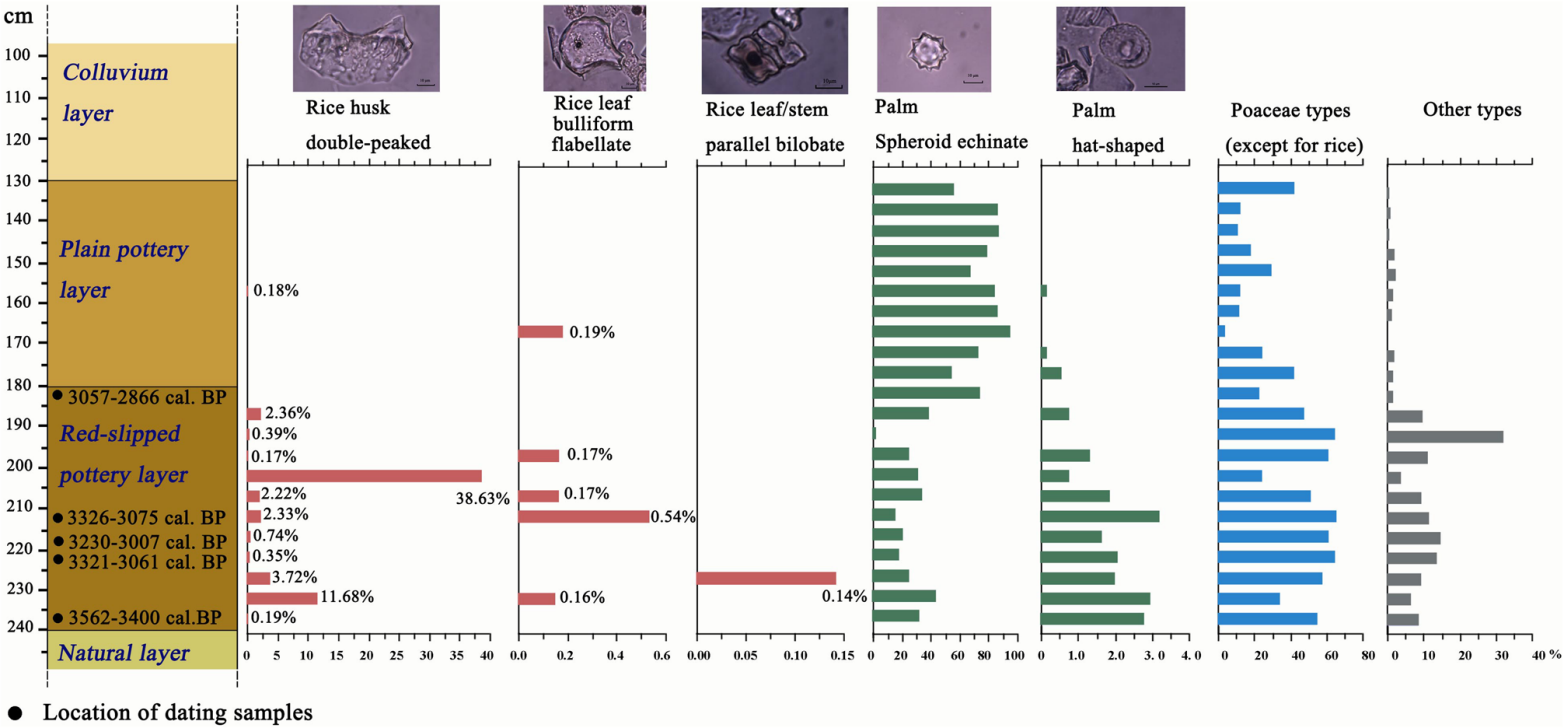
The excavation summary from Minanga Sipakko, showing stratigraphic layers, radiocarbon dates, and proportions of major types of phytoliths (see Table S1 for details and counts) (Created by Z. Deng, using C2 data analysis version 1.7.6 and Adobe Photoshop CC version 19.1.6).

In order to refine the dating of the early pottery horizon at Minanga Sipakko, five new radiocarbon samples from our study confirm the first occupation as early as 3,500 ybp (Figs. 2 and 3; Supplementary Table S1 online). At 240 cm depth, the base of the pottery horizon overlaid a natural riverbank surface, and a sample at 235–240 cm provided a radiocarbon date of 3,562–3,400 ybp. From this base of the pottery horizon, the red-slipped tradition continued upwards through about 190 cm depth of the deposit that encompassed three remarkably similar radiocarbon dates of 3,321-3,061ybp, 3,230-3,007ybp, and 3,326–3,075 ybp. Above 190 cm, small amounts of plain pottery were noticed within the primarily red-slipped assemblage, and then the next sedimentary unit above 185 cm contained rapidly increasing amounts of plain pottery, post-dating 3,057-2,866ybp. In total, the sampled profile provided a sequential record of human activity from at least 3,500 ybp and continuing through 2,800 ybp.

### Identification of rice remains at Minanga Sipakko

The earliest component of the pottery horizon can be distinguished at 240 through 190 cm depth, and therefore the archaeobotanical evidence within this depth range can indicate whether or not rice farming occurred at this time. Nevertheless, our analysis sampled in 5-cm increments throughout all layers, extending upward into the plain pottery contexts post-dating 3,057-2,866 ybp. Within the zone of primary interest, the relevant 5-cm samples were recorded between 235–240 cm and 195–200 cm.

**Figure 4:**
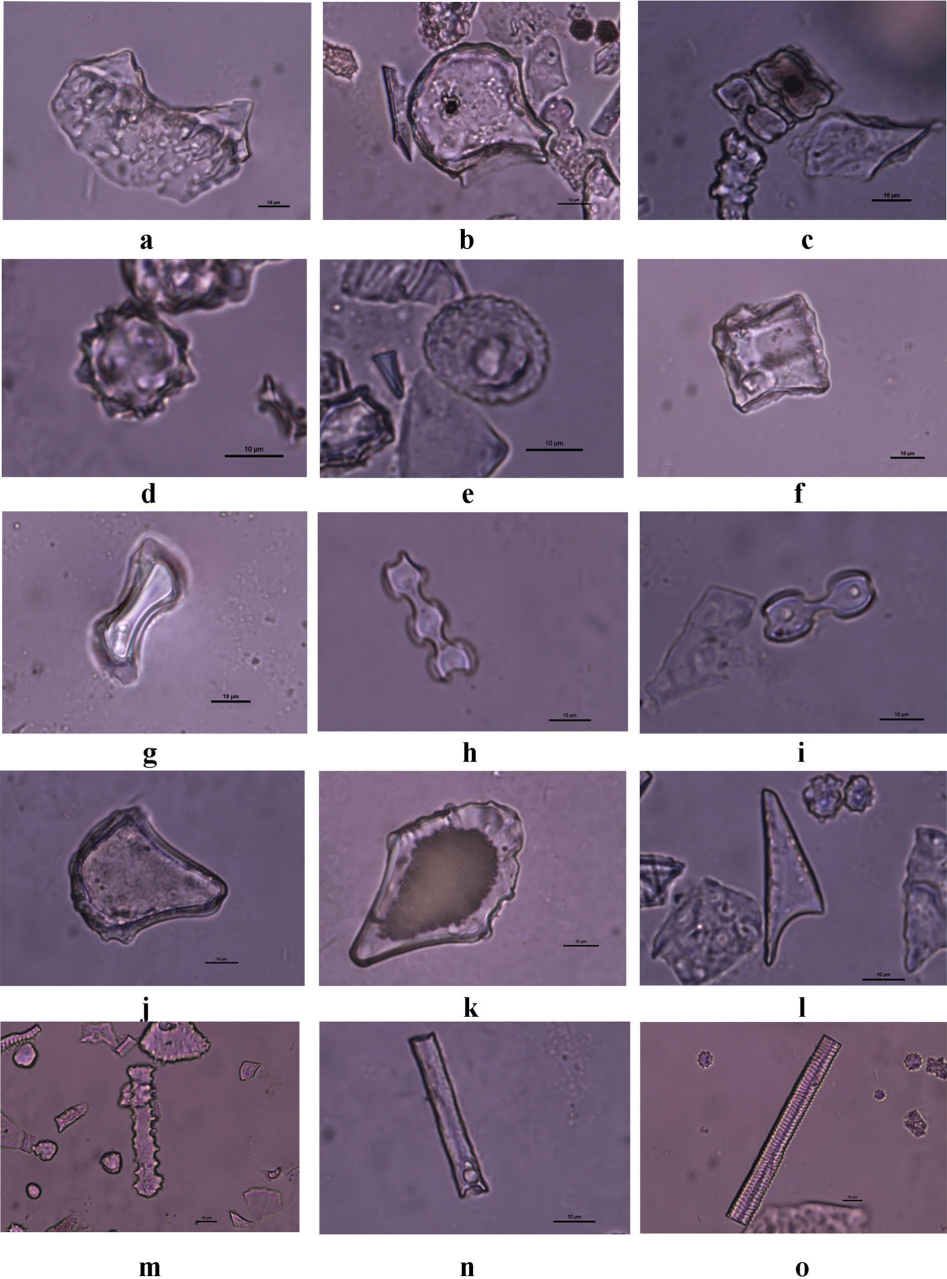
Representative phytolith morphotypes from Minanga Sipakko: (**a**) rice double-peaked, (**b**) rice bulliform flabellate, (**c**) parallel bilobate, (**d**) palm spheroid echinate, (**e**) palm hat-shaped, (**f**) blocky, (**g**) long saddle, (**h**) polylobate, (**i**) bilobate, (**j**) bulliform flabellate, (**k**) bulliform flabellate, (**l**) acute bulbosus, (**m**) elongate dentate, (**n**) elongate entire, (**o**) tracheary annulate (Photographs by Z. Deng).

Rice evidence was found throughout the sampling sequence (see Fig. 3; Supplementary Table S2 online), but the highest concentrations were in the lowest zone with the oldest red-slipped pottery. In this lowest and earliest zone, the most abundant phytoliths were identified as rice plus other grasses, as well as relatively low proportions of palms (Figs. 3 and 4). The pattern was significantly different in the upper zone, post-dating 3,057–2,866 ybp, wherein spheroid echinate palm phytoliths comprised the most abundant category, and rice and the hat-shaped palm phytoliths appeared only in low proportions. Other Poaceae types declined dramatically in the upper layer.

**Figure 5:**
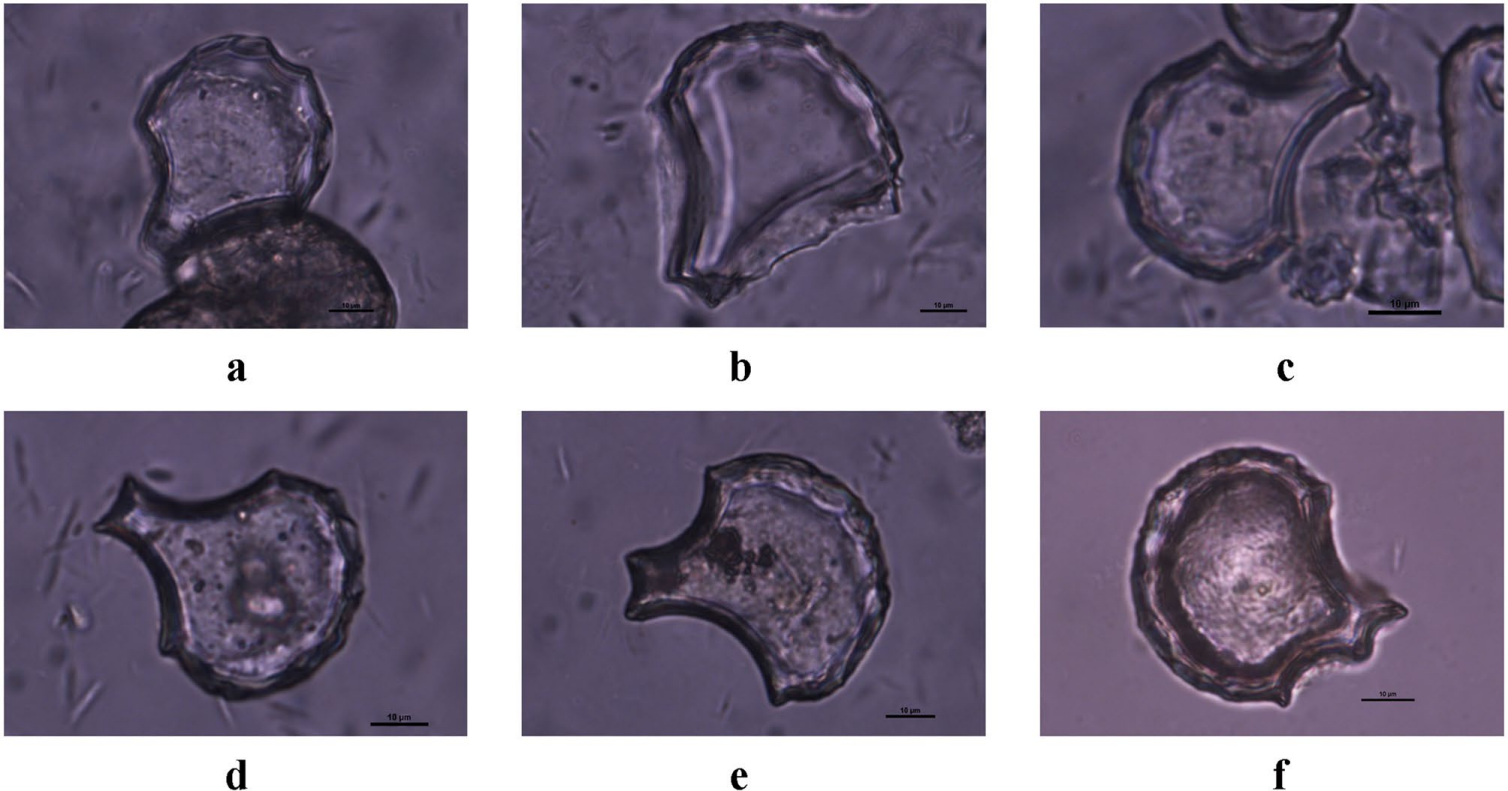
Rice bulliform phytoliths from Minanga Sipakko: (**a**–**c**) with less than 9 scales; (**d**–**f**) with 9 scales or more (Photographs by Z. Deng).

**Figure 6:**
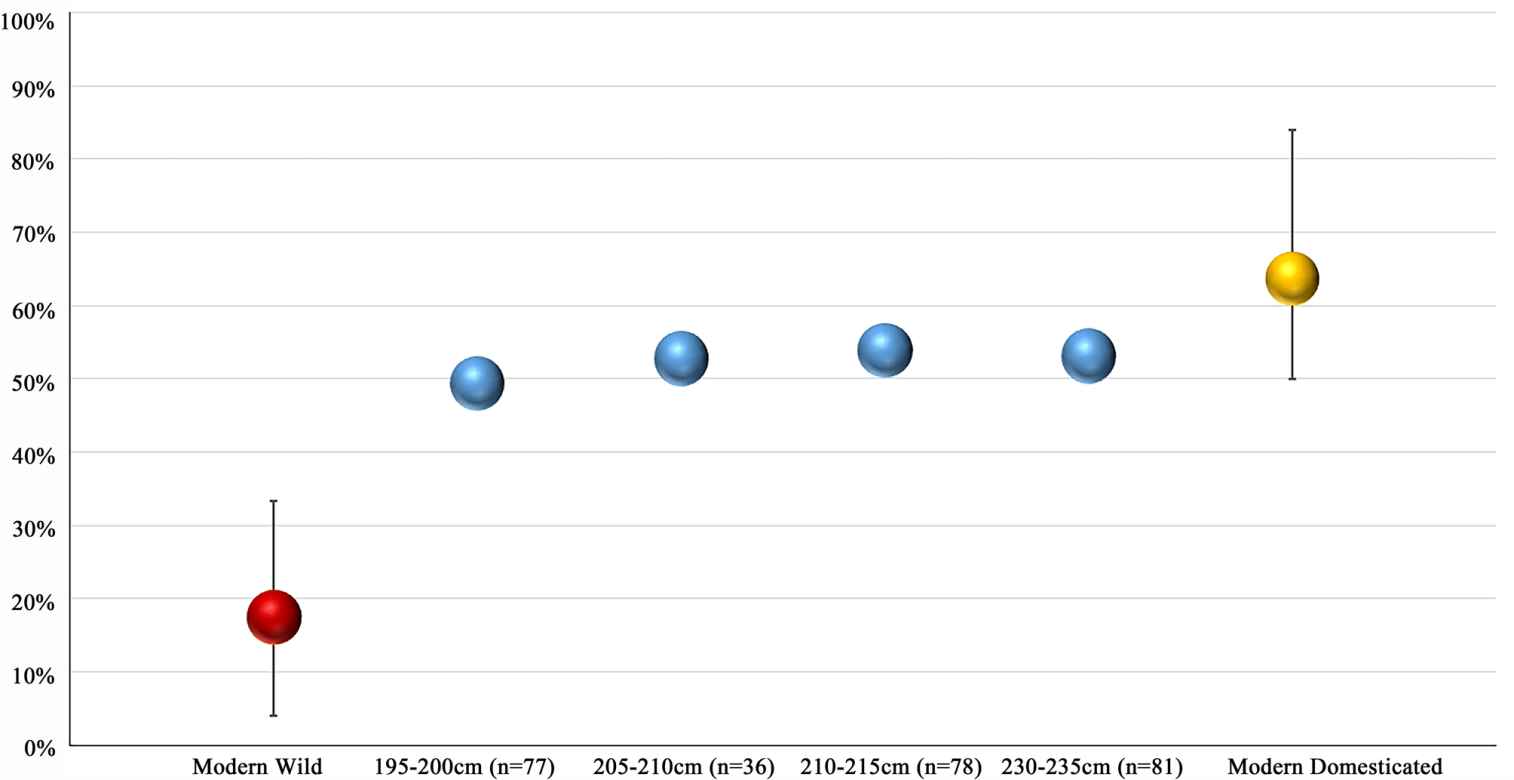
Proportions of rice bulliform phytoliths with at least nine convex edge scales in the Minanga Sipakko samples, compared with modern wild and domesticated samples (Created by Z. Deng, using Microsoft Excel 2019).

Among the rice phytoliths, the leaf bulliform (flabellate) types were scrutinized for the numbers of scales around their convex ends, utilizing the observation that the proportion of bulliform phytoliths with nine or more such scales can identify domesticated (versus wild) varieties^26–28^. In the early occupation layers at Minanga Sipakko, those pre-dating 3,057–2,866 ybp, 50–55% of the leaf bulliform phytoliths consistently displayed nine or more convex-edge scales (Figs. 5 and 6). These consistent proportions are above the 33.33% maximum for convex-edge scales that correlates with wild varieties, and within the expected range for domesticated varieties (Fig. 6; Supplementary Table S3 online). The consistent proportions of these convex-edge scales in all of the early layer samples indicate that domesticated rice was grown under conditions that were consistent throughout the time range of the site.

In addition to the leaf bulliform (flabellate) phytolith morphologies, the rice evidence at Minanga Sipakko suggests the deliberate processing of rice harvests at the site, as reflected in the relative proportions of leaf/stem (bulliform and parallel bilobate) and husk (double-peaked) morphologies (see Fig. 3). According to the crop-processing model based on phytoliths, the early stages of rice processing, such as threshing and winnowing, will produce a much higher proportion of bulliform and bilobate phytoliths than double-peaked phytoliths. At the terminal stage, the phytolith assemblages will be dominated by the double-peaked type, as the end byproduct is mainly rice husk^29^. In most samples from lower layers, the double-peaked type was dominant, especially at depths of 230-235 cm and 200-205 cm, where they accounted respectively for 11.38% and 38.63% of all phytoliths. This suggests that rice processing through to the final stage of husk discard was undertaken in the site during the time of the initial red-slipped pottery horizon.

## Discussion

Prior to this study in Sulawesi, only limited numbers of ancient sites in ISEA had yielded datable but controversial rice-related remains prior to 3,000 ybp, although abundant evidence of rice domestication has been reported as early as 4,800–4,200 ybp from Taiwan^9,24,30^. The absence of compelling evidence in ISEA, until now, has allowed numerous plausible yet ultimately untested notions about the origins of rice farming in the region, as well as about possible links with larger cross-regional population movements.

The new findings from Minanga Sipakko constitute the oldest and the most securely dated evidence of rice farming in Indonesia, in this case co-occurring with the first pottery-bearing horizon and dated as early as 3,562–3,400 ybp. The abruptness of the red-slipped pottery-bearing horizon in the region suggests a sudden appearance of a new cultural tradition.

In ISEA, red-slipped thin-walled pottery has been dated at several sites in Taiwan to as early as 4,800–4,200 ybp. A related pottery tradition thereafter appeared in the northern Philippines around 4,200–4,000 ybp^16,23,24,31,32^. Over the next few centuries, this archaeological signature spread southwards into the central and southern Philippines, eastern Indonesia^33–36^, and the western islands of Remote Oceania^37^. Specifically, the early Minanga Sipakko pottery has shown its closest similarities with the vessel shapes and designs of the early red-slipped pottery assemblages of the Philippines and Taiwan^17,23,24^.

At a larger scale, the spread of this pottery-bearing horizon largely matches the modern geographic distribution of Austronesian-speaking populations in Taiwan, the Philippines, eastern Indonesia, and the western fringe of Remote Oceania. Austronesian was the most widespread language family in the world prior to the colonial era, representing one of the most impressive records of population dispersal in the history of humankind^38,39^. Questions have been debated for nearly a century about the location of the Austronesian homeland, the motives of the ancient migrants, and the nature of the dispersal process^40–46^_._

The results presented here provide general support for the *Farming-language Dispersal Hypothesis* and the closely related *Out of Taiwan Hypothesis* as applied to ISEA^8,9,38,47,48^. These hypotheses refer to Taiwan as the source of Austronesian-speaking rice farmers prior to 4,000 ybp, and then some of their descendants migrated by sea into ISEA in search of new lands. Eventually, these migrants expanded into new territories across more than one third of the globe’s surface, occupying places from Madagascar to Easter Island.

Our conclusions are consistent with recent studies of cranial morphology^49^ and ancient DNA lineages^50^, both suggesting that external biological populations came from East Asia, spread into ISEA after 4,500–4,000 ybp, and became the majority resident groups of ISEA by 3,000 ybp. The linguistic and biological findings appear compatible with the archaeological manifestation of an early pottery-bearing horizon that now can be confirmed as including domesticated rice, at least as far south as Sulawesi.

## Materials and methods

### Sample collection

In 2017, the authors revisited Minanga Sipakko. After cleaning the profile of the east wall of square M2, sediment samples were retrieved from the cultural layer at five-centimeter intervals, starting at the base and continuing upward. In total, 22 samples were collected, starting with the deepest and oldest at a depth of 235–240 cm below modern surface then continuing through the uppermost and latest sample at a depth of 130–135 cm. For each sample, roughly 150 g bulk sediment sample was taken, and around 20 g from each sample then was sub-packaged for phytolith analysis. The remaining samples were sorted under a binocular stereomicroscope at × 15 magnification, and charcoal fragments larger than 0.9 mm were selected for radiocarbon dating. A research permit for the Minanga Sipakko site was granted to T. Simanjuntak (Chief Investigator), A.A. Oktaviana, and B. Hakim to undertake the field research. H.-C. Hung, Z. Deng, and M.T. Carson were invited for laboratory phytolith study, cross-regional comparison, and manuscript preparation.

### Radiocarbon dating

In total, 11 charcoal samples were sent to Beta Analytic Testing Laboratory for accelerator mass spectrometry (AMS) radiocarbon dating. Four samples were too small to yield radiocarbon dates (Beta-508804, 523845, 523846 and 528110), and two were apparently intrusive samples as their radiocarbon dates were older than 43,500 years BP (Beta- 508805 and 523847). Therefore, in total five samples yielded relevant radiocarbon dates. These results were calibrated by OxCal 4.3.2^51^, using the IntCal13 atmospheric curve^52^, and they are presented in Fig. 2 and Supplementary Table S1, together with the previously published dates.

### Phytolith extraction and identification

For each sample, 2 g of sediment was processed to extract phytoliths according to established procedures^53–55^. The weighed samples first were placed inside 50 ml centrifuge tubes and treated with 30% H_2_O_2_. Next, they were left for 12 h for full reaction and to ensure that all organic matter was removed. Three distilled water rinses were performed to remove the residual reagent. Then, carbonate aggregates and certain oxides were removed by adding 15%HCl. After becoming fully reacted, another three distilled water rinses were performed. Phytoliths were floated from the sediments in heavy liquid (ZnBr_2_, density 2.35 g/cm^3^). The suspension with extracted phytoliths was moved into 10 ml centrifuge tubes. Distilled water was added to separate heavy liquid by centrifugation. The concentrated phytoliths then were washed again with 30% ethyl alcohol. In the last step, phytoliths were removed by pipette and mounted on a slide with Canada Balsam.

After air drying, the slides were observed under an optical microscope at 400 × magnification to identify and count all phytolith morphotypes. For each sample, at least 500 phytoliths were identified and recorded according to modern references and published criteria^56–58^. All slides have been browsed thoroughly to search for all well-preserved rice bulliform phytoliths, even after more than 500 phytoliths were recorded. The numbers of scales around the convex end of each rice bulliform phytolith were counted under an optical microscope at 630 × magnification, referring to the published criteria^26,55^.

## Author contributionss

T.S., H.-C. H., Z.D. designed and directed the research; T.S., A.A.O., B.H., and H.-C. H. performed field research; Z.D. analyzed phytolith data. Z.D, H.-C.H. & M.T.C. prepared the manuscript through discussion with T.S.

## Supplementary information

Supplementary file1 (DOCX 30 kb)

Supplementary file2 (XLSX 13 kb)

## Data Availability

All raw data of this study are available in the Supplementary Files (Tables S1–S3), available online with this publication.
