# Supplementary material for: Validating earliest rice farming in the Indonesian Archipelago
Source: Sci Rep. 2020 Jul 3;10:10984. doi: 10.1038/s41598-020-67747-3 (PMC7335082; doi:10.1038/s41598-020-67747-3)
Supplement: Supplementary file 1 — Supplementary file1 (DOCX 30 kb) [file 41598_2020_67747_MOESM1_ESM.docx]

***Supplementary Information for***

**Validating Earliest Rice Farming in the Indonesian Archipelago**

Zhenhua Deng^1,2+*^, Hsiao-chun Hung^3+*^, Mike T. Carson^4^, Adhi Agus Oktaviana^5^,

Budianto Hakim^6^, Truman Simanjuntak^5^

1. Center for the study of Chinese Archaeology, Peking University, Beijing, 100871, China

2. School of Archaeology and Museology, Peking University, Beijing, 100871, China

3. Department of Archaeology and Natural History, Australian National University, Canberra, ACT 2061, Australia

4. Micronesian Area Research Center, University of Guam, Mangilao, Guam 96913, USA

5. Center for Prehistoric and Austronesian Studies, and National Center for Archaeology, Jalan Raya Condet Pejaten 4, Jakarta 12510, Indonesia

6. Balai Arkeologi Makassar, Jl. Pajjaiang No.13, Sudiang Raya, Kota Makassar, Sulawesi Selatan 90242, Indonesia

+First author

* Correspondence and requests for materials should be addressed to Zhenhua Deng (email: zhenhuadeng@pku.edu.cn) or Hsiao-chun Hung (email: hsiao-chun.hung@anu.edu.au).

Table S1. Radiocarbon dates from Minanga Sipakko (All dates are calibrated with OxCal v4.2.4, using the IntCal13 atmospheric curve).

| Square | Depth and sample # | Lab code | Material | Uncalibrated C-14 date (BP) | Calibrated age range (95.4%, cal. BP) | Reference |
| --- | --- | --- | --- | --- | --- | --- |
| M2 | 180–185 cm (19MS-6) | Beta-528107 | Charcoal | 2840±30 | 3057-2866 | this study |
| M2 | 205–210 cm (19MS-3) | Beta-523847 | Charcoal | > 43500 BP | Greater than 46167 |  |
| M2 | 205–210 cm (18MS-2) | Beta-508805 | Charcoal | > 43500 BP | Greater than 46167 |  |
| M2 | 210–215 cm (19MS-8) | Beta-528109 | Charcoal | 3000±30 | 3326-3075 |  |
| M2 | 215–220 cm (19MS-7) | Beta-528108 | Charcoal | 2970±30 | 3230-3007 |  |
| M2 | 220–225 cm (19MS-4) | Beta-523848 | Charcoal | 2980±30 | 3321-3061 |  |
| M2 | 235–240 cm (19MS-5) | Beta-523849 | Charcoal | 3250±30 | 3562-3400 |  |
| TPII | 155–160 cm | P3G-97 | Charcoal | 2570±110 | 2865–2354 | 22 |
| M1 | 170–180 cm | Wk-17981 | Charcoal | 3343±46 | 3691-3461 |  |
| M3 | 155–170 cm | Wk-14651 | Charcoal | 3446±51 | 3838–3583 |  |
| M3 | 220–240 cm | Wk-14652 | Charcoal | 3082±50 | 3400–3161 |  |
| M4 | 200–210 cm | Wk-14653 | Charcoal | 2881±46 | 3158–2878 |  |
| M4 | 250–260 cm | Wk-14654 | Charcoal | 2996±41 | 3340–3040 |  |
| M5 | 170–180 cm | P3G-05 | Charcoal | 3690±160 | 4514–3615 |  |

(Beta: Beta Analytic Testing Laboratory, Florida, USA. P3G: Geology Research and Development Center, Bandung, Indonesia. Wk: Waikato Radiocarbon Dating Laboratory, Hamilton, New Zealand)

Table S2. Original phytolith data from Minanga Sipakko, Indonesia

<insert a separate file>

Table S3. Proportions of rice bulliform phytoliths classified by number of peripheral scales in four phytolith samples from Minanga Sipakko

| Sample Depth  Number of Scales | 195-200cm (n=77) | 205-210cm (n=36) | 210-215cm (n=78) | 230-235cm (n=81) | Total (n=272) |
| --- | --- | --- | --- | --- | --- |
| Four | - | 5.56% | 1.28% | - | 1.10% |
| Five | 3.90% | 2.78% | 2.56% | 3.70% | 3.31% |
| Six | 11.69% | 22.22% | 12.82% | 9.88% | 12.87% |
| Seven | 19.48% | 5.56% | 12.82% | 18.52% | 15.44% |
| Eight | 15.58% | 11.11% | 16.67% | 14.81% | 15.07% |
| Nine | 24.68% | 19.44% | 17.95% | 23.46% | 21.69% |
| Ten | 11.69% | 13.89% | 23.08% | 14.81% | 16.18% |
| Eleven | 11.69% | 16.67% | 8.97% | 8.64% | 10.66% |
| Twelve | 1.30% | - | 1.28% | 4.94% | 2.21% |
| Thirteen | - | 2.78% | 2.56% | 1.23% | 1.47% |
